# Effectiveness of Ng'adakarin Bamocha model in improving access to ante-natal and delivery services among nomadic pastoralist communities of Turkana West and Turkana North Sub-Counties of Kenya

**DOI:** 10.11604/pamj.2015.20.403.4896

**Published:** 2015-04-23

**Authors:** Jillo Ali Jillo, Peter Obonyo Ofware, Susan Njuguna, Wanja Mwaura-Tenambergen

**Affiliations:** 1 Department of Health Systems Management and Medical Education, Kenya Methodist University, Nairobi, Kenya; 2 Amref Health Africa, Kenya Country office, Nairobi, Kenya

**Keywords:** Turkana, Ng´adakarin Bamocha, nomads, pastoralist, skilled delivery services, health facility delivery, traditional birth attendants and container clinics

## Abstract

**Introduction:**

Access to maternal and child health care services among the nomadic pastoralists community in Kenya and African continent in general is unacceptably low. In Turkana, only 18.1% of the women had seen a nurse or a midwife for antenatal care during pregnancy while only 1.3% of pregnant women reported delivery at health facilities in 2005. Ng'adakarin BAMOCHA model, based on migratory routes of the Turkana pastoralists and container clinics was adopted in 2007 to improve access to maternal and child health services by the nomads.

**Methods:**

A cross-sectional study design was used to establish the effectiveness of Ng'adakarin BAMOCHA model on accessibility and uptake of ante-natal care and delivery services. A total of 360 households and 400 households were interviewed for pre-intervention and post-intervention respectively. The study compared the pre-intervention and post-intervention findings. Structured questionnaires and focus group discussion were used for data collection.

**Results:**

There was no improvement in the fourth ante-natal care visits between pre-intervention and post-intervention groups at 119(51.5%) and 111(41.9%) respectively (p < 0.05). Knowledge of the community on the importance of ANC visits improved from 60%-72% with significance level of p < 0.05. There was a significant increase 6%-17% of deliveries under a skilled health worker (p < 0.05). TBA assisted deliveries increased from 7.5%- 20.2% with a p < 0.05. There was significant reduction in home deliveries from 89.5%-79.5% with a p < 0.05.

**Conclusion:**

The Ng'adakarin Bamocha model had a positive effect on the improving maternal health care among the nomadic pastoralist community in Turkana.

## Introduction

Access to health is one of the key human rights. This is enshrined in various international treaties and agreements such as Millennium development goals. In Kenya, health was identified as key pillar in developing the nation in vision 2030 and further enshrined in Kenya health policy 2012 - 2030. The key elements of rights to health are the accessibility of services, availability of health services, acceptability of health services and quality of health care (WHO, 2007) [1].

### Rural and nomadic pastoralist community health care access

Access to health care overall is a challenge to rural residents, who have a lower proportion of the population insured, a greater difficulty in traveling to primary, preventative, prenatal, and emergency care providers, and less diversity in health care resources to choose from. Rural residents are left without these services, increasing the physical barriers to quality and timely healthcare. The geographic variances in access lead to the conclusion that different strategies to address health disparities will have to be considered for rural regions (Jones Ian et.al., 2011) [2].

The disparities in health care access are more pronounced among the nomadic pastoralist communities of Sub-Saharan Africa. Pastoralists migrate periodically with their herds to maximally exploit scarce resources (pasture and water), which they need for their animals and themselves and which are dispersed in time and space. It seems likely that this seasonal movement is an important determinant of nomadic people's health (Sheik-Mohamed & Velema, 1999) [3].

Nomadic populations are defined as communities of people that temporarily or permanently move their residence and occupational activities from one location to the other. Nomads include nomadic hunter and gatherers, pastoralists and peri-pathetic communities (i.e. groups of people moving around settled populations and offering a craft or trade). Pastoralists can be further differentiated into a) trans-humans (nomadic groups migrating regularly between two grazing areas along well-defined routes), b) pastoralists migrating along conventional routes but also moving into different areas each year and semi-pastoralists with semi-sedentary residence and mobility patterns (Okeibunor et.al., 2013) [4].

Nomads are often at a disadvantage for receiving health care. In Somalia, for example, the national health plan 1985-1990 recognized that 90% of nomads were out of the reach of the national health services. This scenario is replicated in Turkana County where close to 80% of the population are nomadic pastoralists (AMREF, 2007) [5]. One study conducted among the settled and nomadic Rendille in Kenya revealed that all children over 12 months of age in Korr village had full immunization coverage which is in contrast to the nomadic community of the same area immunization coverage of zero (Nathan & Rot, 1996) [6].

A study among the Fulani nomads of Nigeria concludes that the nomads are currently disadvantaged in the provision of health interventions due to physical and psychological distance that exists between them and the sedentary population as well as health officials in the areas (Dao & Brieger, 1995) [7]. Ailou (Ailou, 2010) [8] argued that it is possible to organize PHC services for nomads. The services should be capable of mobility matching that of the community they serve. They should establish seasonal circuits in accordance with the local patterns of population movements. Similarly, Al-Omar and Bin Saeed, (1999) [9] emphasized the need to set PHC programmes for nomadic populations, especially in countries with limited resources and large nomadic communities. Here, it is reasoned that health services for the nomads could be organized with their full involvement in the planning and implementation. Ailou, (2010) [8] further believes that despite intervention from the government, partners and other development agencies most of the interventions for nomadic communities are either not cost-effective or inefficient. Landmark series on child and neonatal survival suggests that the high mortality persists despite low cost solution being known and that almost 60-70% of these deaths could be prevented by making these interventions widely available (Darmstadt et.al., 1999) [10]. It is worth noting that these areas have the same basic ecological and socio-economic characteristics with the study area. The intervention measures intended to reduce the child morbidity and mortality are pegged upon prevention and treatment. Prevention measures suggested include: Outreach services (effective ANC) clinic based care include skilled maternal and immediate newborn care and extra care of low birth weights. Treatment measures mentioned include oral rehydration therapy, appropriate antibiotics for pneumonia, Zinc for diarrhea, appropriate anti-malarials and emergency obstetric care.

### Antenatal care uptake

Kenya's Service provision assessment of 2010 establishes that all pregnancy are at risk of developing complications and as such to ensure good health outcome for both mother and baby pregnant women should have access to preventive interventions, early diagnosis and treatment and emergency care when needed. Studies have shown the benefits related to routine Ante-natal care for pregnant women including early management of dangers signs, preparation for delivery through individualized birth plans, provision of supplements and vaccination including tetanus toxoid vaccine ANC is designed to promote healthy behaviors and preparedness during pregnancy, childbirth and delivery (NCAPD, 2010; WHO 2003) [11, 12].

To provide proper management of pregnant women's health and in lieu of the cost and other barriers involved in access of health, WHO recommends at least 4 ante-natal visits during pregnancy (WHO, 2002) [13] There is a trend of decline in proportion of women who make four or more ANC visits during pregnancy in Kenya from 52% in 2003 to 47% in 2008 (KNBS, 2010) [14]. There is also discrepancy between geographical location of clients with rural (43.1%) and urban (59.9%) women attending 4th ANC (KNBS, 2010) [14].

There is close association between the level of knowledge of the community on the importance of ante-natal care and its relationship with safe delivery. Therefore most intervention target capacity building of the community as basis of improvement in ANC attendance during pregnancy. Ediau et al. (2013) [15] showed that In Uganda, Kitgum, having introduced intervention through health care workers capacity building, provision of medical supplies and community health education, the odds of a pregnant woman attending ANC increased to 11.4 compared to pre-intervention.

Other studies have also shown the importance of demographic variables in influencing uptake of ante-natal care. There is also close association between the knowledge of the community and level of education attained. van Eijk et al. (2006) [16] showed that women who do not attend ANC were more likely to have less than 8 years of education and a low socio-economic status. The same trend of women with low level of education, low knowledge of Ante-natal care and low socio-economic status not accessing ANC is depicted by Nisar & White (2003) [17].

### Delivery services utilization

Millennium development goal number five calls for improvement in maternal health with targets of reducing maternal mortality ratio (MMR) by three quarters by 2015 and by improving access to reproductive health. Though review of progress towards goal 5 shows global improvement (Lozarno, 2011) [18]. In Kenya more women are dying from pregnancy related causes in 2009 compared to 2003 with MMR of 488 and 412 per 100 000 live births respectively (KNBS, 2010) [14]. 80% of maternal deaths are due to severe bleeding infections, high blood pressure during pregnancy and unsafe abortion (Sheik-Mohamed & Velema, 1999) [3]. In Kenya, the rate of skilled delivery stands at 44% while delivery at health facilities is at 43% (KNBS, 2010) [14]. In rural areas and hard to reach areas like Turkana the rate is further diminished to 8% skilled delivery (AMREF, 2007) [5]. However, the key causes of deaths can be prevented or managed with proper continuum of care during pregnancy and delivery (NCPD, 2013) [19]. As discussed under ante-natal care, interventions to improve delivery targets knowledge of the community on skilled and health facility deliveries. In Zambia, Ensor et al. (2014) [20] showed that community based intervention can lead to improvement of knowledge of community on importance of delivery and other maternal child health indicators. The community based intervention included training of health care workers, health education to communities and involvement of men in women health. The study showed improvement of heath facilities deliveries from 49% at pre-intervention to 75% post intervention. In another study, Rojahn (2010) [21] shows the importance of community based approaches (similar in strategy to Ng'adakarin Bamocha model) in improving maternal and child health. The study concludes that while the importance of skilled delivery and facility-based services for maternal and newborn care cannot be denied, there is sufficient evidence to scale up community-based care through packages which can be delivered by a range of community-based workers.

### Intervention

Unlike other nomadic communities in Kenya, Turkana pastoralists migrate in groups with the entire family. Livestock movements are organized into homesteads of single extended families. A group of Awis forms an Adakar (livestock camp), which consists of pastoralist families that have agreed to move together under a recognized leader in pursuit of pasture, water and security. Groups of Adakar are known as Adakarin, while ‘Ng'adakarin’ means ‘together’. The Adakar is headed by a council of elders representing all herdsmen. Migration patterns of different Ng'adakarin are composed of 40 to 100 households, each under the leadership of the Ng'adakarin heads (AMREF, 2007) [5].

The Ng'adakarin Bamocha intervention included introduction of twenty feet long freight container clinics. The container clinics were strategically located along migratory routes to ensure the Adakarin are always within walking distance to Level I health services. The container clinics are only operational when the communities have migrated to within its vicinity and when the community moves away, the next closest clinic is opened. The clinics are operated by a trained community nurse and an Adakar Community Health Worker. They provide curative services, immunization, referral to Level II health facilities and community education, as well as acting as a restocking point for the ACHWs drug kits. The container clinics are equipped with a fridge, VHF communication radio powered by solar, basic drugs and laboratory test kits. The container is modified into two rooms, with one room operating as a clinic and the second providing accommodation for the attending nurse.

Community mobilization was the foundation to the model from both the outset, and throughout the life of the project. Leaders then engaged their respective *Adakarin*to sensitize and mobilize the wider community. Community mobilization is used to raise awareness, increase health knowledge and demand for health care and enable the *Adakar* community to take an active role in their own health care (AMREF, 2007) [5]. The ACHWs are identified and selected by *Adakar* members are trained to offer maternal child health services and information, diagnose and treat minor illnesses with drug kits, as well as to educate the community on the prevention and control of prevalent diseases.

## Methods

The study covered two divisions each in Turkana North and Turkana West Sub-Counties: Lokichoggio, Oropoi, Kaaleng and Kibish. The study design was a cross-sectional study for pre-intervention and post-intervention population with intention of establishing the changes in access to delivery services. The study population comprised of mothers with children aged 0 ≤ 23 months and Health care workers. The study population was drawn from the 17 Ng'adakarin covered by the intervention in four divisions. Convenience sampling was employed at Ng'adakarin level (17 Adakarin), resulting into eight study clusters. At the Adakar level simple random sampling was employed was used to select study respondents who are women with children aged below 23 months. 360 women were interviewed in pre-intervention while 400 women were interviewed for post-intervention. Four FGDs were conducted with women and men in the four divisions of intervention. Four key informants (1 chief, 1 member of local Community Based Organization, Sub-County Nursing Officer and Sub-County Medical officer of Health) were interviewed.

Quantitative data was entered using Ms Access sheet. Data was managed and analyzed using SPSS version 21. Qualitative data was entered and grouped using Ms Excel. A bivariate analysis was carried out to relate socio-demographic variables to antenatal care use and other health-seeking behavior of the mother for her child between 0-23 months. Chi square test was used as appropriate with p-value set at the 0.05 level.

Study limitation: the study covers the nomadic pastoralist community that make up 80% of the population therefore excluding sedentary community. The study used convenience sampling to select half of Ng'adakarin reached with intervention because of inaccessibility of some of Adakar to be studied as they migrate, even, across international borders in search of pastures and water for their livestock.

## Results

The mean age for pre-intervention and post-intervention respondents was 20.1 (Standard deviation 4.9) and 28.5 (standard deviation 4.8) respectively. M most respondents in the pre-intervention 275(76.4%) and post-intervention 307 (69.7%) were between the ages of 20-34. The level of education for both pre-intervention and post-intervention respondents was low with 327 (90.8%) pre-intervention and 382 (86.6%) post-intervention reporting to have not attained any formal education. Just over three-quarters 290 (80.6%) and 338 (76.8%) of respondents in the pre-intervention and post- intervention were Christians respectively. In general study population for the pre-intervention and post- intervention groups were from similar and comparable demographic background and therefore the results could be compared. Complete characteristics are shown in Table 1.

**Table 1:** Demographic information of respondents

Socio-demographic characteristics	Respondents	
	Pre-intervention N=360 (%)	Post-intervention N=400 (%)
**Age groups**		
15-19 years	10 (2.8)	32 (7.9)
20-24 years	75 (20.8)	114 (28.6)
25-29 years	92 (25.3)	97 (24.3)
30-34 years	109 (30.3)	67 (16.8)
35-39 years	39 (10.8)	46 (11.6)
40-44 years	30 (8.3)	24 (6.1)
45-49 years	7 (1.9)	19 (4.8)
Mean	28.1 years	28.5 years
Std. deviation	4.9 years	4.8 Years
**Education**		
None	327 (90.8)	346 (86.6)
Primary	17 (4.7)	37 (9.3)
Secondary+	11 (3.1)	8 (2.0)
Not stated	5 (1.4)	8 (2.0)
**Religion**		
Christians	290 (80.6)	307 (76.8)
Muslim	2 (0.6)	4 (1.1)
Traditional	49 (13.6)	38 (9.5)
Other	7 (1.9)	6 (1.4)
Not stated	12 (3.3)	45 (11.2)

In the pre-intervention assessment, 148 (64.2%) respondents indicated that they attended ANC for their last pregnancy at least once compared 165 (62.5%) who attended ANC at least once in the post-intervention assessment. Considering that the respondents who indicated that they attended ANC in the pre-intervention, 6 (2.5%) could not give an indication of number of times they attended and hence the percentage attended at least once could be exactly the same for both the pre-survey and the post-survey. Table 2 shows the number of ANC attendance.

**Table 2:** Ante-natal care attendance

Number ANC Visits reported during last pregnancy	Pre-intervention *(n = 231)* Frequency (%)	Post-intervention *(n = 265)* Frequency (%)	*p-value*
Once	33 (14.3)	32 (12.1)	0.12
Twice	31 (13.4)	65 (24.5)	0.02*
Three times	42 (18.2)	57 (21.5)	0.05*
Four times	119 (51.5)	111 (41.9)	0.04*
Not stated	6 (2.5)	-	-

Of the respondents who indicated that they attended ANC in the pre-intervention survey, 33(14.3%) attended only once compared to 32(12.1%) who attended only once in the post-intervention assessment (p > 0.05), 31(13.4%) attended twice compared to 65(24.5%) who attended twice in the post-intervention which was significantly different (p < 0.05), 42 (18.2%) attended thrice as compared to 57(21.5%) who attended thrice in the post intervention and is statistically different (p < 0.05) and 119 (51.5%) attended four times or more as compared to 111 (41.9%) who attended four times or more in the post-intervention study and is statistically different (p < 0.05). results indicate that number of women attending ANC more than once (i.e. twice and thrice) increased significantly while those attending four or more times decreased significantly.

**Figure 1:**
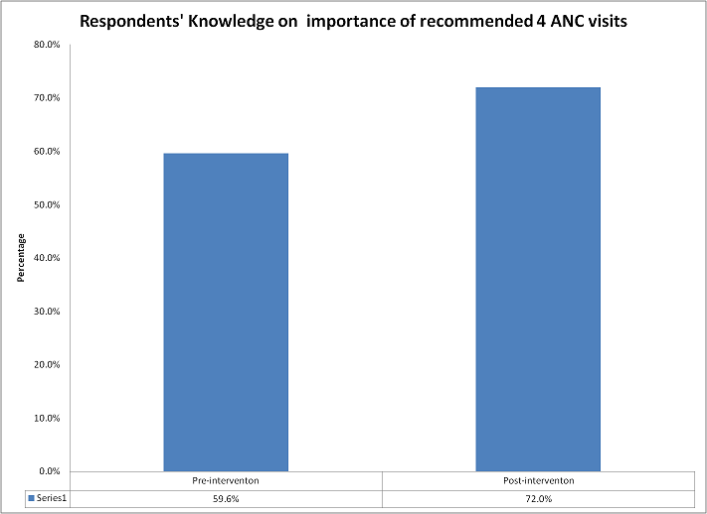
Respondents’ knowledge on recommended at least 4 antenatal visits

*Knowledge on recommended at least 4 ANC* When the respondents were asked whether they knew that a pregnant woman is supposed to attend ANC at least 4 times before they deliver, in the pre-intervention survey, 131 (59.6%) indicated that they knew about this while in the post-intervention survey, 191 (72.0%) indicated that they knew that a pregnant mother should attend at least 4 times before delivery. There is a statistical difference between this knowledge at the pre-intervention period and the post-intervention period (p < 0.05). Figure 1 shows the proportion of respondents pre-intervention and post intervention who are aware of the recommended at least 4 visits to health facilities before delivery.

### 

#### Delivery services

The pre-intervention survey established that only 22 (6.2%) of the deliveries were done in health facilities while 317 (89.5%) of deliveries took place at home through the help of friends, relatives, traditional birth attendants and/or by themselves. This is in comparison to 66 (16.5%) of deliveries in the post-intervention survey, which took place in a health facility compared to 318 (79.5%), which took place at home. This indicates significant increase in health facility delivery (p < 0.05) where it has almost tripled between the time of the pre-intervention survey and the post intervention assessment. The summary of places of delivery for the respondent for the last pregnancy is shown in (Table 2).

The pre-intervention survey established that 20(5.6%) of the births were delivered by a trained birth attendant compared to 65(17.7%) in the post-intervention survey which shows a significant increased in births assisted by trained medical practitioner (p < 0.05). The number of births attended by a TBA and by relatives and friends also significantly increased from 27(7.5%) and 142(39.4%) in the pre-intervention to 74(20.2%) and 170(46.0%) in the post-intervention. On the other hand, the percentage of self-deliveries greatly reduced from 162(45.0%) in the pre-intervention survey to 59 (16.1%) in the post-intervention survey. The number of assisted deliveries is summarized in (Table 3).

**Table 3:** Delivery services

	Pre-intervention N (%)	Post-intervention N (%)	P-value
**Places of Delivery**			
Home	317 (89.5%)	318 (79.5%)	0.04*
Away from home	15 (4.2%)	10 (2.5%)	0.12
Health facility	22 (6.2%)	66 (16.5%)	0.03*
Other/Not stated	-	5 (1.3%)	-
**Assistance During Delivery**			
Skilled attendants (Midwife/Nurse/doctor)	20 (5.6%)	65 (17.7%)	0.03*
TBA	27 (7.5%)	74 (20.2%)	0.03*
Relatives/friends	142 (39.4%)	168 (46.0%)	0.06
Self	162 (45.0%)	59 (16.1%)	0.01**
Not stated	9 (2.5%)		-

Findings from focused group discussions and Key informant interviews were consistent with quantitative analysis deliveries. Respondents reported Cost (medical and transport cost involved), walking distance to delivery health facilities and lack of capacity of nearest facility (including container clinic) in terms of infrastructure (delivery rooms) and personnel. It is interesting that culture is mentioned as a barrier to utilization of delivery services though most of the respondents agree that health information available to them has tampered its influence.

## Discussion

The demographic result shows that the two samples of the population for pre intervention and post intervention has similar attributes in terms of distribution in age, educational status and religious affiliation. This makes the two samples comparable without introducing sample bias.

Attendance of ANC remained almost the same for pre-intervention and post intervention at around. However, the trend in decrease of women attending at least 4 ANC needs to be addressed. The proportion of women attending at least 4 ante-natal care clinics during pregnancy is similar to national average of 47% (KNBS, 2010) [14]. The trend of decrease in numbers of women accessing the recommended 4th Ante-natal care is also shown by the Kenya demographic health surveys in 2003 and 2009 (KNBS, 2010) [14] where there was decline from 52% to 47%. Other studies have shown that uptake of ante-natal care is dependent on level of education of the community and household (van Eijk et.al.,2006; Nisar & White 2003) [16, 17]. Considering that both pre-intervention and post-intervention groups have high proportion of women with no formal education, the uptake of ANC is commendable in the study areas. The results also showed gaps between knowledge of the respondents on importance of recommended at least four ante-natal visitations and uptake of Ante-natal care. This can be attributed to the lifestyle of the community where they move away from established health facilities including container clinics in search of waters and pasture for their animals. The community knowledge improvement components of the model yielded results in knowledge on Ante-natal care. This is especially important as several studies have shown the positive relationship between levels of knowledge of the community and uptake of antenatal care. van Eijk et.al. (2006) [16] showed that the odds of women attending ANC increases with knowledge and level of education. Similar conclusion is arrived at by Ediau et.al. (2013) [15]; where the odds of uptake of ANC increases by 11.4 for community reached with interventions including community health education. This shows that the Ng'adakarin Bamocha model has laid down the foundation for improvement in uptake of ANC through improvement in knowledge of the community despite low prevalence of 4th antenatal care at the time of the study.

Place of delivery and delivery under skilled care are important in determining the health outcome of both mother and baby. Skilled personnel are important in managing complications during pregnancy while health facility delivery contributes to hygienic conditions of delivery (Wagstaff & Claeson, 2004) [22]. The improvement in Health facility deliveries and deliveries under supervision of skilled personnel is indicative of effectiveness of the Ng'adakarin BAMOCHA model. The improvement is attributed to health information provided by Health care workers at health facilities along migratory routes and by the Adakar community health workers. The health facilities were renovated and expanded to provide room for delivery. Further improvement in skilled delivery results from health care workers supervising deliveries at home. These findings are consistent with results findings (Ailou, 2010) [8] where there was significant improvement in delivery under skilled care after intervention through implementation of community strategy. However the skilled delivery and health facility deliveries coverage in Turkana is still way below the national average of 44% and 43% respectively (AMREF, 2007) [5].

Traditional birth attendants continue to play an important role in deliveries in Kenya and Africa at large with high proportion of deliveries conducted by TBAs [5, 9]. The study established significant increase in TBAs assisted deliveries despite their lack of capacity to manage top-five causes of maternal mortality (Darmstadt et.al., 1999) [10]. The communities’ preference for TBA deliveries is due to their proximity in the community, low cost (transport cost and medical cost) and long walking distances to health facilities (NCAPD, 2010; WHO 2003) [11, 12]. Low rate of skilled delivery can also be attributed to capacity of container clinics to provide only referral services for deliveries despite close proximity to the migratory pastoralist community.

## Conclusion

The study results shows that the *Ng'adakarin* BAMOCHA approach based on community owned and community lifestyle tailor-made interventions led to improvement in skilled and health facility deliveries. The Ng'adakarin BAMOCHA model strengthens referrals between the community (ACHWs and container clinics) and Level Two and above services. The study recommends recruitment of more health care workers in line with community health strategy and their training to effectively deliver health education including the importance of ante-natal care attendance. The study also recommends that Health partners and the Ministry of health at County level to consider establishing maternity waiting homes to allow pregnant women from the migratory community a resting place as they wait to deliver.
